# Novel formulations ameliorate osteoarthritis in rats by inhibiting inflammation and oxidative stress

**DOI:** 10.1002/fsn3.4407

**Published:** 2024-08-13

**Authors:** Fusun Erten, Oguzhan Ozdemir, Muhammed Tokmak, Ali Said Durmus, Ibrahim Hanifi Ozercan, Abhijeet Morde, Muralidhara Padigaru, Kazim Sahin

**Affiliations:** ^1^ Department of Veterinary Medicine, Pertek Sakine Genc Vocational School Munzur University Tunceli Turkey; ^2^ Department of Veterinary Science, Technical Sciences Vocational School Batman University Batman Turkey; ^3^ Department of Animal Nutrition, Faculty of Veterinary Medicine Firat University Elazig Turkey; ^4^ Department of Surgery, Faculty of Veterinary Medicine Firat University Elazig Turkey; ^5^ Department of Pathology, Faculty of Medicine Firat University Elazig Turkey; ^6^ Research and Development OmniActive Health Technologies Mumbai India

**Keywords:** anti‐inflammatory, inflammation, osteoarthritis, oxidative stress, plant‐based formulations

## Abstract

The study tested new oral plant‐based formulations (F) on rats with monosodium iodoacetate (MIA)‐induced osteoarthritis, measuring inflammation, antioxidant levels, paw size, stride, and analyzing knee joint images. Fifty‐six female Sprague Dawley rats were allocated into 8 groups: (1) Control, (2) MIA (OA induced with MIA), (3) MIA + F1 [curcuminoids+gingerols+acetyl‐11‐keto‐β boswellic acid (AKBA)], (4) MIA + F2 (curcuminoids+Withania glycosides+AKBA), (5) MIA + F3 (curcuminoids+total withanolides+AKBA), (6) MIA + F4 (curcuminoids, AKBA), (7) MIA + UCII (type II collagen), and (8) MIA + GCHON (Glucosamine Chondroitin). Treatments F1 to F4 reduced right joint diameter and improved stride length and paw area in OA rats. Despite improvements with treatments F1 to F4, there was no significant difference between these groups (*p* > .05). In OA animals, F1 to F4 treatments decreased MDA levels and increased antioxidant enzymes activities (*p* < .001). This was done by reducing levels of inflammatory markers and enzymes like IL‐1β, IL‐6, MMP‐8, TNF‐α, CRP, COMP, and LOX‐5, while increasing the anti‐inflammatory cytokine IL‐10. In conclusion, these plant‐based treatments significantly reduced osteoarthritis severity, slowed disease progression by reducing inflammation, and protected joints from damage, showing a protective effect in rats with induced osteoarthritis, likely due to their anti‐inflammatory and antioxidant properties.

## Graphical abstract

Treatments F1 (curcuminoids + gingerols + AKBA) to F4 (curcuminoids + AKBA) improved joint diameter, stride length, and paw area in OA rats. They significantly reduced MDA levels and increased antioxidant enzyme activities by lowering inflammatory markers such as IL‐1β, IL‐6, MMP‐8, TNF‐α, CRP, COMP, and LOX‐5, while increase the anti‐inflammatory cytokine IL‐10. These findings suggest that F1 to F4 effectively alleviates oxidative stress and inflammation in OA.

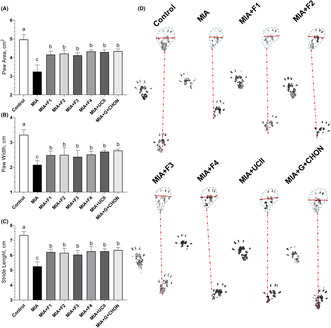

## 1 INTRODUCTION

Global life expectancy has been accompanied by a corresponding increase in osteoarthritis (OA) prevalence. As the most common musculoskeletal disease, especially in the elderly, OA causes functional decline, pain, and loss of quality of life (Pereira et al., 2015; Sacitharan, 2019). An estimated 30.8 million adults in the United States and 300 million people worldwide live with OA, which is estimated to cost 303 billion dollars annually in medical expenses and lost earnings (Abramoff & Caldera, 2020; McCoy, 2015). OA, which most commonly affects the joints, including the knees, hands, hips, and spine, is a leading cause of movement impairment in older people (Xia et al., 2014). In contrast to earlier paradigms, OA is now understood to be a low‐grade inflammatory disease that affects entire joints and progresses to the gradual destruction of the articular cartilage, synovial inflammation, subchondral bone remodeling, and osteophyte formation, with meniscus and ligament involvement (Liu et al., 2023). Non‐steroidal anti‐inflammatory medicines (NSAIDs), which only temporarily relieve pain and have no effect on reversing cartilage degeneration, are among the treatments now available for OA. These drugs have known toxicities and adverse effects; therefore, the search for new therapeutic techniques for the prevention and treatment of OA continues, as well as the search for a better understanding of the molecular and cellular pathways and how they relate to joint tissues. Developing new therapeutic approaches with few or no side effects is urgently needed (Ansari et al., 2020).

Many studies have documented abnormally high TNF‐α, IL‐6, and IL‐1 levels in OA patients as essential factors contributing to cartilage loss (Lee et al., 2021; Wang & He, 2018). These inflammatory factors worsen knee OA by activating corresponding pathways and promoting the release of inflammatory mediators (Wang et al., 2020). IL‐1β is recognized as the primary inducer of OA. By triggering the expression of matrix metalloproteinases (MMPs), IL‐1β might encourage the cleavage of the cartilage matrix, which causes the extracellular matrix, including collagen II and aggrecan, to degrade during the pathogenesis of OA (Ohzono et al., 2023). The production and release of inflammatory mediators and catabolic agents, including iNOS, COX‐2, PGE2, TNF‐α, and MMPs that contribute to chondrocyte dysfunction and the breakdown of the extracellular matrix, are supported by the release of IL‐1β (Fei et al., 2019).

A major challenge in studying osteoarthritis (OA) as it naturally develops in patients is that clinical symptoms often do not accurately mirror the molecular events and structural changes occurring within the joint. This is because such symptoms typically become apparent later in the disease process. Numerous animal models, either surgically or chemically induced, can address this problem (McCoy, 2015). In this study, osteoarthritis (OA) was induced in rats through the injection of monosodium iodoacetate (MIA). This approach offers a quick, reliable, and cost‐effective means of inducing OA. It yields a pathology similar to human OA in many respects while circumventing the potential sensitivities and risks associated with surgery (Rebai et al., 2020).

Medicinal plants are crucial in treating diseases, particularly since no specific drug has yet been identified to combat osteoarthritis (OA) effectively. Therefore, the screening and mechanistic exploration of herbal medicines remain highly significant. In this context, the present study focused on the diverse health benefits of curcumin, a hydrophobic polyphenol derived from the rhizomes of *Curcuma longa* (Yavarpour‐Bali et al., 2019). Gingerol, the active component of ginger, which is recognized for its significant medicinal properties, was also incorporated into the prepared formulations. Studies have reported that mitogen‐activated protein kinase modulates proinflammatory mediators (TNF‐α and COX‐2) (Nafees et al., 2021). In addition, Boswellia, another ingredient in the formulation, has many pharmacological properties, and acetyl‐11‐keto‐β boswellic acid (AKBA) has been accepted as its main active ingredient. AKBA has been shown to have multiple beneficial effects on immune systems, including the modulation of cytokine levels (such as ILs and TNF‐α) and the suppression of reactive oxygen species (ROS) formation (Catanzaro et al., 2015). *Withania somnifera*, also known as Ashwagandha, is highly valued in traditional medicine and is commonly consumed as a functional food for its myriad therapeutic benefits. The medicinal properties of this plant are primarily attributed to its active components, withanolides. Over 40 different withanolides, encompassing new compounds, have been extracted from the plant. Notably, some withanolide glycosides have been identified with β‐D glucopyranose attached at either the C‐3 or C‐27 position (Lee et al., 2022). Numerous medicinal plants, including Boswellia *serrata* and *Curcuma domestica*, along with their secondary metabolites, demonstrate activities against osteoarthritis (OA) (Wang et al., 2022). In this study, multi‐component formulations labeled as OA health treatments, OAHT F1–F4, were developed. These formulations comprised various plant‐based active compounds known for their beneficial properties, such as turmeric, ginger, *B. serrata*, and Ashwagandha. These were then evaluated in a rat model of OA induced by MIA. The objective of the study was to investigate the effects of novel orally administered plant‐based formulations on serum inflammation and biochemical parameters, the activity of antioxidant enzymes, levels of malondialdehyde (MDA), dimensions of paw areas and diameters, and stride lengths. Additionally, the study encompassed the evaluation of radiographic and histopathologic images of the knee joint to assess the supplements' impact on osteoarthritis.

## 2 MATERIALS AND METHODS

### 2.1 Animals and experimental design

Fifty‐six female Sprague Dawley rats (age: 8 weeks, mean weight: 180 ± 20 g), which were obtained from the Firat University Experimental Research Center (FUDAM), were used in the study. The rats were kept in an environment at a constant temperature (23 ± 2°C), humidity (55% ± 10%), and light cycle (12/12 h light–dark), with access to food and drink throughout the experiment. The study was conducted in compliance with the accepted ethical standards for the care and use of laboratory animals as specified in the regulations of the European Economic Community (EEC, 1986) and approved by the Animal Ethics Committee of Firat University (E‐97132852‐60401.02‐32,814).

Rats were randomly divided into eight groups, with seven in each group:

1. Control: Rats received saline without any induction of OA.
2. MIA: A single intra‐articular injection of MIA‐induced OA, but no additional treatment was administered.
3. MIA + F1: OA was induced by MIA, and the rats were treated with a combination of curcuminoids, gingerols, and acetyl‐11‐keto‐β boswellic acid (AKBA).
4. MIA + F2: OA was induced, and rats were treated with curcuminoids, withania (Ashwagandha) glycosides, and AKBA.
5. MIA + F3: OA was induced, and rats were treated with curcuminoids, total withanolides (from Withania *somnifera* or Ashwagandha), and AKBA.
6. MIA + F4: OA was induced, and the treatment was curcuminoids combined with AKBA.
7. MIA + UCII: OA was induced, and the treatment was undenatured type II collagen.
8. MIA + G + CHON: OA was induced, and the rats were treated with a combination formula that is representative of the “Move Free Advanced Glucosamine Chondroitin” supplement. This includes glucosamine hydrochloride, chondroitin sulfate, hyaluronic acid, and calcium fructoborate.

The formulations were supplied by OmniActive Health Technologies (Mumbai, India). The doses of formulations are shown in Table 1. Doses of formulations were determined based on the Human Equivalent Dose for Drug Development (Shin et al., 2010). Recommended daily human doses (HED) were converted to animal doses based on body surface area. A conversion factor 6.17 was used to convert human doses to rat doses.

**TABLE 1:** Preparation of the formulations with human and animal equivalent doses.

No	Formulations	Ingredients	Animal dose (mg/kg BW)
1	F1	Curcumionids + Gingerols + AKBA	17
2	F2	Curcumionids + Withania Glycosides + AKBA	25
3	F3	Curcumionids +Total Withanolides + AKBA	18
4	F4	Curcumionids + AKBA	15
5	UCII	Undenatured type II collagen	4
6	Move Free Advanced G+ CHON	Glucosamine Hydrochloride, Chondroitin Sulfate, Hyaluronic Acid, Calcium Fructoborate	197

Abbreviations: AKBA, acetyl‐11‐keto‐β boswellic acid; CHON, Chondroitin Sulfate, Hyaluronic Acid and Calcium Fructoborate; G. Glucosamine Hydrochloride; OAHT, Osteoarthritis Health Formulation; UCII, undenatured type II collagen.

The OA rat model was performed as previously described (Jeong et al., 2017; Lu et al., 2018). The rats were anesthetized with xylazine (10 mg/kg) and ketamine hydrochloride (50 mg/kg). The right knee was shaved and cleaned with 70% alcohol. A 0.3 mL insulin syringe with a 29 G needle was used to inject 1.0 mg of MIA (Sigma, St. Louis, MO, USA) into the right knee joint through the infrapatellar ligament. Saline 50 μL was injected into the control group. Two weeks after the MIA injection, treatments were administered orally for 4 weeks after being dissolved in 1 mL of saline. Every other day on alternating days, all rats were monitored to measure knee joint swelling. Rats were euthanized 4 weeks after treatment, and blood and knee joint samples were collected for analysis.

### 2.2 Serum biochemical parameters

Using a portable automated chemistry analyzer (Samsung LABGEO PT10V, Samsung Electronics Co., Suwon, Korea), serum creatinine, blood urea nitrogen (BUN), total protein (TP), aspartate aminotransferase (AST), and total bilirubin (TBIL) were examined. Following the manufacturer's instructions, serum levels of TNF‐α, IL‐1β, IL‐6, cartilage oligomeric matrix protein (COMP), and C‐reactive protein (CRP) were measured using commercially available enzyme‐linked immunosorbent assay (ELISA) kits (Cayman Chemical, Ann Arbor, MI, USA).

The activities of catalase (CAT), glutathione peroxidase (GSH‐Px), and superoxide dismutase (SOD) were measured using commercially available kits. (Cayman Chemical, Ann Arbor, MI, USA) according to the manufacturer's instructions. For MDA analysis, the Shimadzu UV–vis SPD‐10 AVP detector at a flow rate was made from a CTO‐10 AS VP column, and 30 mM KH_2_PO_4_ and methanol (82.5: 17.5, v/v, pH 3.6) was used (Shimadzu, Japan) at 1.2 mL/min. The column effluent was monitored at 250 nm.

### 2.3 Joint swelling (edema) measurement

Every other day, all rats were inspected to measure the edema in their knee joints. Under anesthesia, three measurements of the right knee joint thickness were made using an electronic digital caliper, and the average in mm was determined. The rats were also clinically assessed for pain and inflammation.

### 2.4 Gait test

After having their hind legs covered with ink, the rats were enticed to walk on a track of white paper 60 cm long and 7 cm broad to a dark room at the end of the route. When the exam was finished, the paper was scanned at 300 dpi. The following factors were used to calculate paw circumference: paw area (cm^2^), the distance between the first and fifth toes, paw width (cm), the distance between steps taken by the same hind paw, stride length (cm), and horizontal distance between toes. The distance between the soles of the left and right paws (cm), the distance between the third toe and the heel (cm), and the paw angle (°) were provided as well. ImageJ software (version 1.43u, National Institutes of Health, Bethesda, MD, US) was used to measure the steps.

### 2.5 Calculation of the Kellgren–Lawrence score and cartilage assessment

Experienced radiologists assessed each rat's level of OA and the severity at each joint according to the Kellgren–Lawrence grading system (Kellgren & Lawrence, 1957; Table 2). An experienced senior surgeon blinded to the research groups evaluated the degree of articular cartilage injury for each joint compartment using the Mankin system (Mankin et al., 1971; Table 3).

**TABLE 2:** Kellgren–Lawrence scoring system (Kellgren & Lawrence, 1957).

Stage	Radiologic findings
0	None
1	Doubtful: Suspicious narrowing of the joint space and possible osteophyte formation
2	Minimal: Definite osteophyte and possible narrowing of the joint space
3	Moderate: Numerous moderate osteophytes, definite narrowing of the joint space, some sclerosis, and possible deformity of the bone ends
4	Severe: Large osteophytes, marked narrowing of the joint space, sclerosis, and deformity of the bone ends

**TABLE 3:** Cartilage evaluation according to the Mankin system (Mankin et al., 1971).

Criteria	Score	Histological finding
Structure	0	Smooth intact surface
	1	Slight surface irregularities
	2	Pannus/surface fibrillation
	3	Clefts into the transitional zone
	4	Clefts into the radial zone
	5	Clefts into the calcified zone
	6	Total disorganization
Cells	0	Uniform cell distribution
	1	Diffuse cell proliferation
	2	Cell clustering
	3	Cell loss
Tidemark integrity	0	Intact
	1	Vascularity

### 2.6 Histological evaluations

Histological alterations were assessed to determine the treatment effect on cartilage degradation in the knee joints of the rats with OA. Each knee joint was removed from the euthanized rats, preserved in 10% formalin for 24 h at 4°C, and decalcified for 4 days at 4°C with 5% hydrochloric acid (Fischer et al., 2008). The samples were decalcified, dried in graded acetone, and then embedded in paraffin. Hematoxylin–eosin (H&E) staining was applied to sections (thickness, 2–3 μm) for 5 and 3 min, respectively. An expert histopathologist, unaware of the research groups, took digital images of the histologic preparations under a microscope.

### 2.7 Western blotting

Western blotting was used to assess the amount of joint tissue proteins (IL‐1β, IL‐6, IL‐10, TNF‐α, COMP, and MMP‐8) in articular cartilage samples (Yabas et al., 2021). Joint tissue samples were first homogenized, followed by electrophoresis, and 20 μg of protein was transferred to a nitrocellulose membrane. Primary antibodies (IL‐1β, IL‐6, IL‐10, TNF‐α, COMP, and MMP‐8; Abcam, Cambridge, UK) were diluted and incubated with the membranes, followed by incubation with a secondary antibody that had been peroxidase‐conjugated. Finally, the image analysis system (Image J, National Institute of Health, Bethesda, MD, US) was used to assess the relative intensities of the bands seen with diaminobenzidine solution. Data are expressed as a percentage.

### 2.8 Statistical analyses

The G * Power program (Version 3.1.9.2) was used to calculate the sample size for the study with an alpha error of 0.05 and an 85% power (Cohen, 1988; Faul et al., 2007). A Shapiro–Wilk test was used to implement conformism to normality from the preliminaries of the parametric tests, and a Levene test was used to assess the homogeneity of the variances. The analysis of variance (ANOVA) test was employed to identify group differences, and the post hoc Tukey test was utilized for multiple group comparisons. The radiologic and histopathologic scores were examined for nonparametric data using Kruskal–Wallis and Mann–Whitney U tests. The threshold for statistical significance was set at *p* < .05.

## 3 RESULTS

### 3.1 Biochemical parameters

The evaluation of the safety profile of the supplements involved measuring serum biochemical parameters. It was found that inducing osteoarthritis (OA) in subjects using MIA and treating them with herbal formulations did not significantly affect key serum biochemical parameters, such as creatinine, TP, BUN, AST, ALT, and TBIL (*p* > .05; Table 4).

**TABLE 4:** Effects of different joint health formulations on serum biochemical parameters in monosodium iodoacetate (MIA) induced osteoarthritis rats.

Parameters	Groups								*p*
	Control	MIA	MIA + F1	MIA + F2	MIA + F3	MIA + F4	MIA + UCII	MIA + G + CHON	
ALT, U/L	91.29 ± 6.63	90.43 ± 5.38	92.29 ± 6.24	91.14 ± 7.63	88.29 ± 7.25	93.43 ± 6.65	89.57 ± 7.85	90.43 ± 7.28	0.916
AST, U/L	187.43 ± 10.67	186.71 ± 9.64	193.14 ± 17.3	188.57 ± 14.4	191.14 ± 9.01	184.57 ± 33.49	190.00 ± 13.01	189.86 ± 24.79	0.993
BUN, mg/dL	16.57 ± 2.4	15.26 ± 2.33	16.71 ± 2.30	17.03 ± 1.78	16.63 ± 2.1	16.33 ± 2.26	16.03 ± 0.68	16.81 ± 1.29	0.783
Creatinine, mg/dL	0.50 ± 0.09	0.49 ± 0.08	0.48 ± 0.14	0.50 ± 0.09	0.51 ± 0.07	0.50 ± 0.11	0.49 ± 0.09	0.50 ± 0.08	0.999
TP, g/dL	6.91 ± 0.30	7.09 ± 0.32	7.09 ± 0.41	6.91 ± 0.38	7.00 ± 0.47	6.90 ± 0.16	6.91 ± 0.42	7.03 ± 0.55	0.956
TBIL, mg/dL	0.26 ± 0.05	0.25 ± 0.01	0.24 ± 0.02	0.26 ± 0.03	0.26 ± 0.03	0.25 ± 0.04	0.25 ± 0.03	0.27 ± 0.02	0.810

Note: Hyaluronic Acid and Calcium Fructoborate. *p* < .05; ANOVA and Tukey's post hoc test. Mean values of parameters are demonstrated with ± standard deviation. Abbreviations: ALT, Alanine aminotransferase; AST, Aspartate aminotransferase; BUN, Blood urea nitrogen; CHON, Chondroitin Sulfate; G, Glucosamine Hydrochloride; MIA, monosodium iodoacetate; TBIL, Total Bilirubin; TP, Total protein; UCII, undenatured type II collagen.

### 3.2 Gait test

**FIGURE 1:**
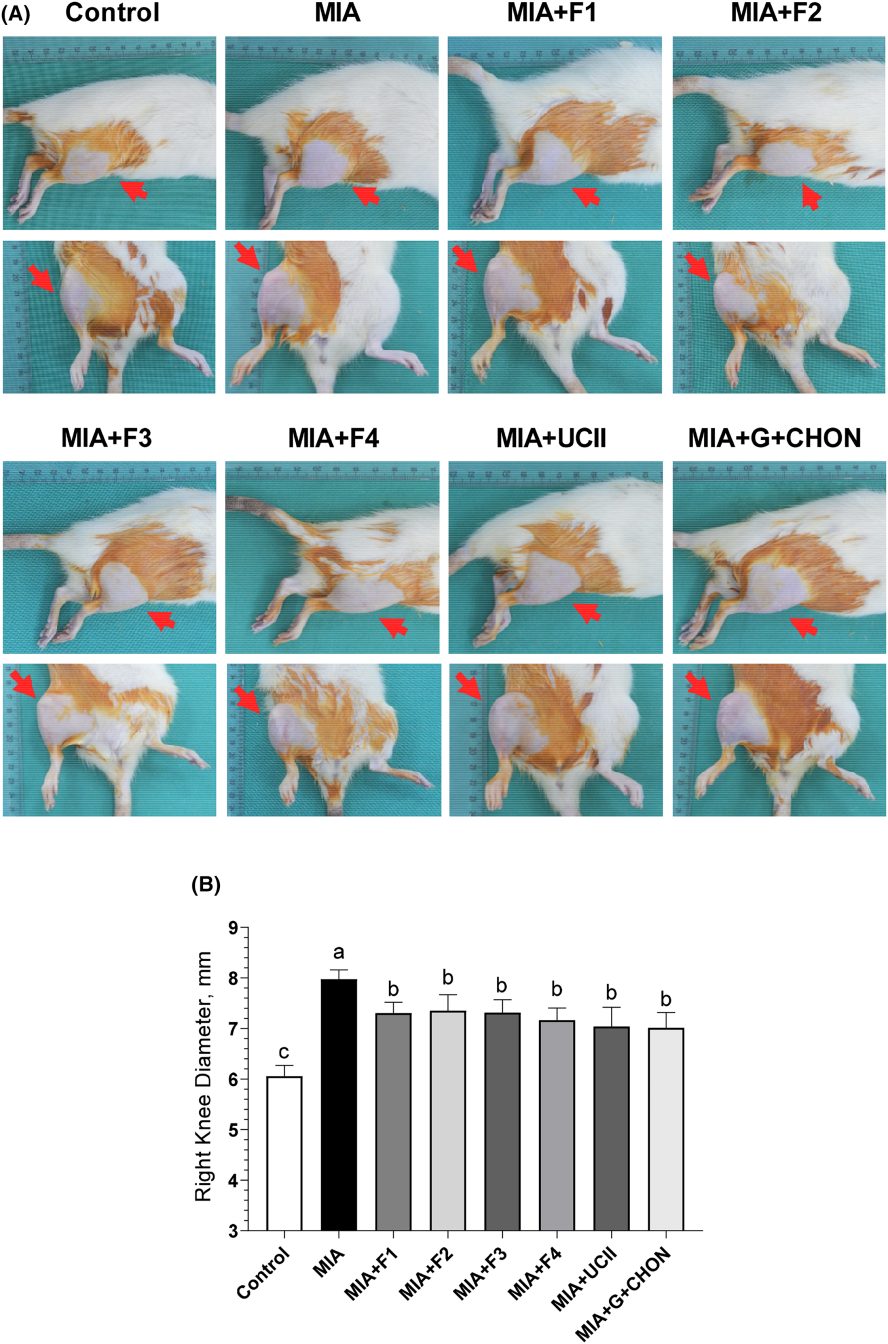
Effects of different OAHT formulations on rat knee swelling (A) and knee joint diameter (B) associated with monosodium iodoacetate (MIA)‐induced OA. The bars and error lines point out the mean and standard deviation. ANOVA and Tukey's post hoc test were performed for statistical comparison. Different letters (a, b, and c) above the bars indicate statistical differences among the groups (*p* < .05). F1:Curcuminoids+Gingerols+acetyl‐11‐keto‐β boswellic acid (AKBA); F2: Curcuminoids+Withania Glycosides+ AKBA; F3: Curcuminoids+Total Withanolides+AKBA; F4:Curcuminoids+AKBA; UCII: Undenatured type II collagen; G + CHON: Glucosamine Hydrochloride, Chondroitin Sulfate, Hyaluronic Acid, Calcium Fructoborate.

**FIGURE 2:**
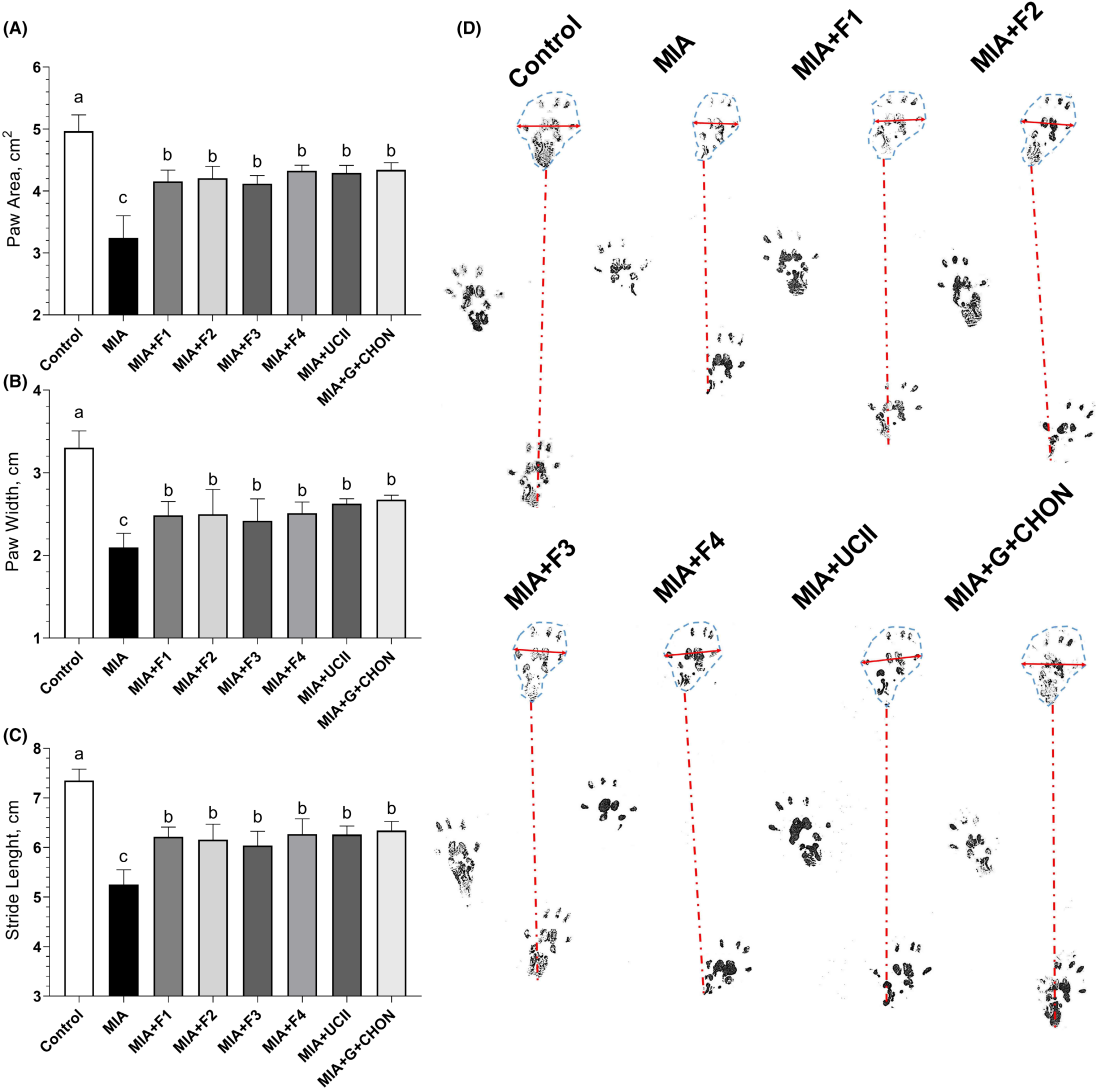
Effects of different OAHT formulations on rat paw area (A), paw width (B), and stride length (C) in monosodium iodoacetate (MIA)‐induced OA. Representative measures are shown (D). The bars and error lines point out the mean and standard deviation. ANOVA and Tukey's post hoc test were performed for statistical comparison. Different letters (a, b, and c) above the bars indicate statistical differences among the groups (*p* < .05). F1: Curcuminoids+Gingerols+acetyl‐11‐keto‐β boswellic acid (AKBA); F2: Curcuminoids+Withania Glycosides+ AKBA; F3: Curcuminoids+Total Withanolides+AKBA; F4:Curcuminoids+AKBA; UCII: Undenatured type II collagen; G + CHON: Glucosamine Hydrochloride, Chondroitin Sulfate, Hyaluronic Acid, Calcium Fructoborate.

To compare the knee swelling induced by MIA, the right joint diameters of the rats were measured. Rats with MIA‐induced osteoarthritis (OA) exhibited significantly larger right knee joint diameters compared to the control group (Figure 1). However, when the F1 to F4 formulations were administered for 4 weeks, they led to a substantial reduction in right joint swelling in comparison to the OA untreated group (*p* < .0001). Notably, there was no significant difference between the F1 to F4 groups and the UCII and G + CHON groups (*p* > .05; Figure 1A,B). The investigation into the effectiveness of supplements in addressing gait abnormalities induced by osteoarthritis (OA) in rats revealed that MIA injection decreased stride length and claw area. However, the supplementation with these formulations partially reversed these changes, as indicated by the significance (*p* < .0001, Figure 2A–D).

**FIGURE 3:**
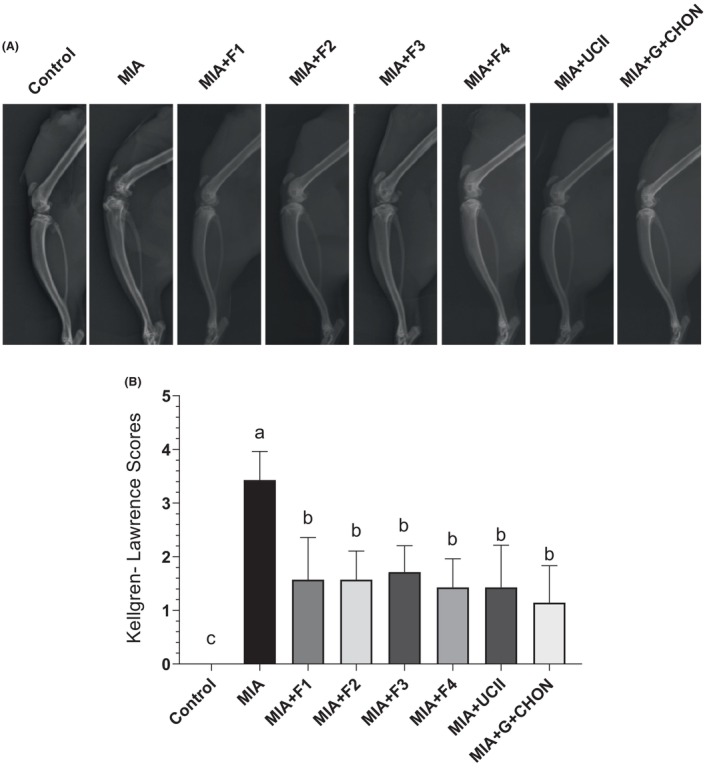
Effects of different OAHT formulations on the rat knee joint with monosodium iodoacetate (MIA)‐induced OA. Representative radiographic images are shown (A). Mean values of Kellgren–Lawrence scores are demonstrated (B) with standard deviation. Kruskal–Wallis and uncorrected Dunn's multiple comparisons test were performed for statistical comparison. Different letters (a, b, and c) above the bars indicate statistical differences among the groups (*p* < .05). F1:Curcuminoids+Gingerols+ acetyl‐11‐keto‐β boswellic acid (AKBA); F2: Curcuminoids+Withania Glycosides+ AKBA; F3: Curcuminoids+Total Withanolides+AKBA; F4:Curcuminoids+AKBA; UCII: Undenatured type II collagen; G + CHON: Glucosamine Hydrochloride, Chondroitin Sulfate, Hyaluronic Acid, Calcium Fructoborate.

The radiographic characteristics of the knee joint in rats with MIA‐induced OA were assessed using the Kellgren–Lawrence rating method to determine the effects of the formulations on the pathogenesis of OA. Compared to control animals, those with MIA‐induced OA had noticeable pathological alterations in the knee joint on X‐ray imaging. Interestingly, all formulations were observed to considerably minimize abnormalities caused by MIA in the rats (Figure 3A,B).

**FIGURE 4:**
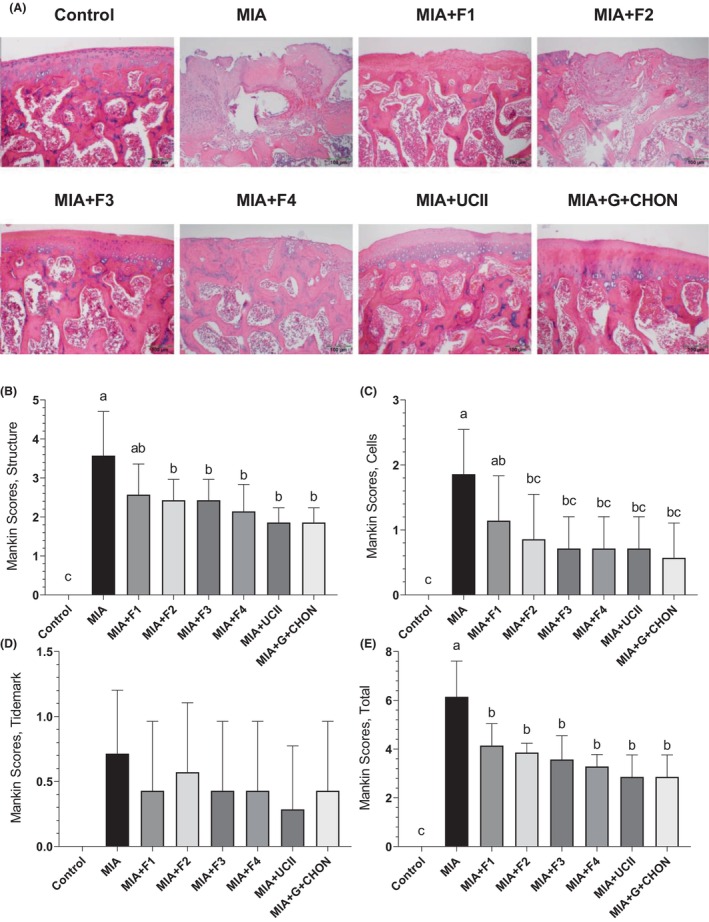
Effects of different OAHT formulations on rat knee joint histopathology associated with monosodium iodoacetate (MIA)‐induced OA. Representative hematoxylin–eosin stained joint cartilages are shown (A). Mean values of structure (B), cells (C), tidemark (D), and total Mankin scores (E) are demonstrated with standard deviation. Kruskal–Wallis and uncorrected Dunn's multiple comparisons test were performed for statistical comparison. Different letters (a, b, and c) above the bars indicate statistical differences among the groups (*p* < .05). F1: Curcuminoids+Gingerols+ acetyl‐11‐keto‐β boswellic acid (AKBA); F2: Curcuminoids+Withania Glycosides+ AKBA; F3: Curcuminoids+Total Withanolides+AKBA; F4:Curcuminoids+AKBA; UCII: Undenatured type II collagen; G + CHON: Glucosamine Hydrochloride, Chondroitin Sulfate, Hyaluronic Acid, Calcium Fructoborate.

Histopathological analysis following H&E staining revealed that rats with MIA‐induced OA exhibited significantly greater inflammatory cell infiltration and more severe joint structural deterioration than the control group. However, supplementation with the F1–F4 formulations effectively mitigated these abnormal features, as depicted in Figure 4A–E. In summary, the results indicate that the supplements improved the outcomes of OA, demonstrating similar efficacy to the UCII and G + CHON groups used as a safety comparison, with all groups showing statistical significance (*p* < .0001 for all).

### 3.3 Inflammatory mediators

**FIGURE 5:**
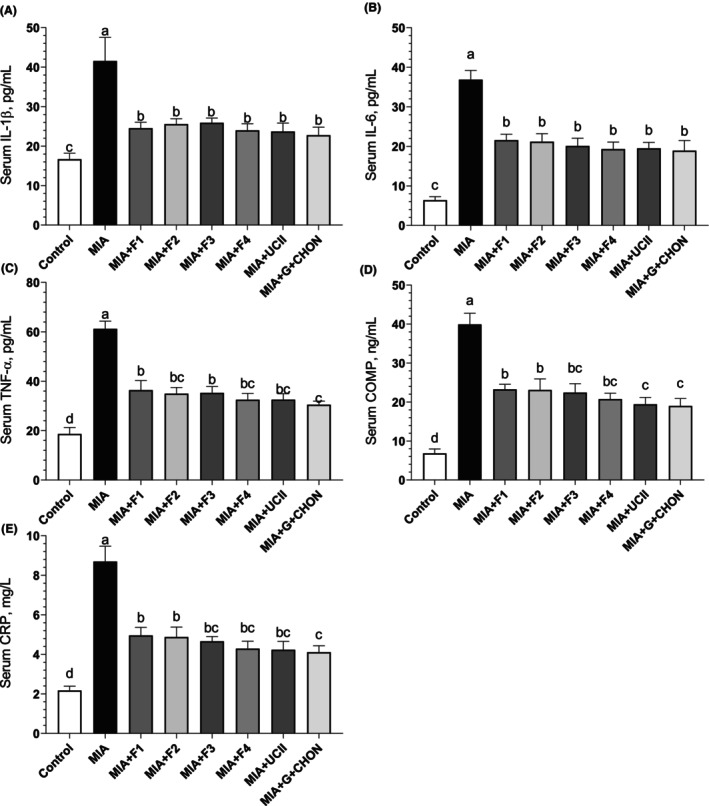
Effects of different OAHT formulations on serum IL‐β (A), IL‐6 (B), TNF‐α (C), COMP (D), and CRP (E) levels in rats with monosodium iodoacetate (MIA)‐induced OA. The bars and error lines point out the mean and standard deviation. ANOVA and Tukey's post hoc test were performed for statistical comparison. Different letters (a, b, and c) above the bars indicate statistical differences among the study groups (*p* < .05). IL‐1β, interleukin‐1β; IL‐6, interleukin‐6; TNF‐ α, tumor necrosis factor α; COMP, cartilage oligomeric matrix protein; CRP, C‐reactive protein. F1: Curcuminoids+Gingerols+ acetyl‐11‐keto‐β boswellic acid (AKBA); F2: Curcuminoids+Withania Glycosides+ AKBA; F3: Curcuminoids+Total Withanolides+AKBA; F4:Curcuminoids+AKBA; UCII: Undenatured type II collagen; G + CHON: Glucosamine Hydrochloride, Chondroitin Sulfate, Hyaluronic Acid, Calcium Fructoborate.

Serum CRP and COMP levels were elevated in rats with MIA‐induced OA but were considerably lowered when OAHT was added to the diet (*p* < .0001; Figure 5D,E). Compared to the OA group, the COMP level decreased by 47.99% and the CRP level by 50.65% in the group receiving curcuminoids + AKBA supplementation. However, when the formulations were compared, no significant difference was found between the levels of COMP and CRP (*p* > .05). In addition, a significant increase in serum levels of the proinflammatory cytokines IL‐1β, IL‐6, and TNF‐α was detected in rats with MIA‐induced OA compared to control animals (*p* < .0001). Furthermore, using F1 to F4 effectively decreased the amounts of cytokines that cause inflammation (IL‐1β, IL‐6, and TNF‐α) compared with the OA untreated group (*p* < .0001; Figure 5A–C). The most effective formulation, curcuminoids + AKBA (MIA + F4), reduced serum IL‐1β levels by 42.30%, IL‐6 levels by 47.63%, and TNF‐α levels by 46.9% compared to the OA untreated group. There was no significant difference between the groups supplemented with F1–F4 (*p* > .05).

### 3.4 Oxidative stress

**FIGURE 6:**
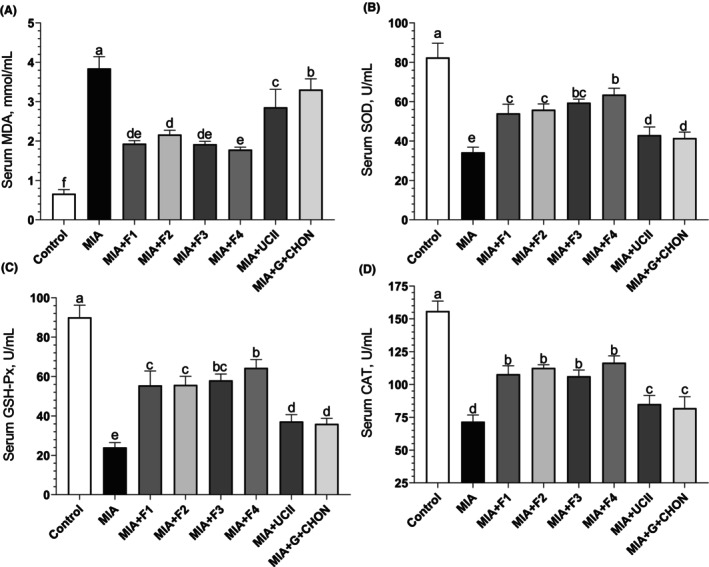
Effects of different OAHT formulations on serum MDA (A), SOD (B), GSH‐Px (C), and CAT (D) levels in rats with monosodium iodoacetate (MIA)‐induced OA. The bars and error lines point out the mean and standard deviation. ANOVA and Tukey's post hoc test were performed for statistical comparison. Different letters (a, b, and c) above the bars indicate statistical differences among the groups (*p* < .05). MDA, malondialdehyde; SOD, superoxide dismutase; GSH‐Px, glutathione peroxidase; CAT, catalase. F1: Curcuminoids+Gingerols+ acetyl‐11‐keto‐β boswellic acid (AKBA); F2: Curcuminoids+Withania Glycosides+ AKBA; F3: Curcuminoids+Total Withanolides+AKBA; F4:Curcuminoids+AKBA; UCII: Undenatured type II collagen; G + CHON: Glucosamine Hydrochloride, Chondroitin Sulfate, Hyaluronic Acid, Calcium Fructoborate.

Given that oxidative stress is crucial to the development of OA (Ansari et al., 2020), it was predicted that OAHT could also positively affect the oxidative stress signal in rats with OA. To assess the condition, serum MDA levels were measured as a sign of oxidative stress. Rats receiving MIA to generate OA had MDA levels 82.76% higher than the control group (*p* < .0001). The administration of next‐generation F1 to F4 partially reversed this increase. In rats treated with curcuminoids + AKBA (MIA + F4), the most effective of the health formulations, MDA levels decreased by 53.66% compared to the OA untreated group (Figure 6A). However, it was determined that the addition of gingerols (F1) or total withanolides (F3) did not have a significant effect on MDA levels compared to the curcuminoids + AKBA (F1) (*p* > .05). Additionally, when concentrations of antioxidant enzymes SOD, CAT, and GSH‐Px were assessed, there was a decrease in serum antioxidant levels in rats with MIA‐induced OA (*p* < .0001, Figure 6B–D). The reduction in antioxidant enzyme levels was partially corrected with the supplementation of F1–F4 formulations. Compared to the MIA‐induced OA group, the most effective formulation was curcuminoids + AKBA (MIA + F4) (Figure 6B–D). These data suggest that the developed F1‐F4 formulations can play an important role in regulating oxidative stress markers in the pathogenesis of OA.

### 3.5 IL‐1β, IL‐6, IL‐10, TNF‐α, COMP, and MMP‐8 levels

**FIGURE 7:**
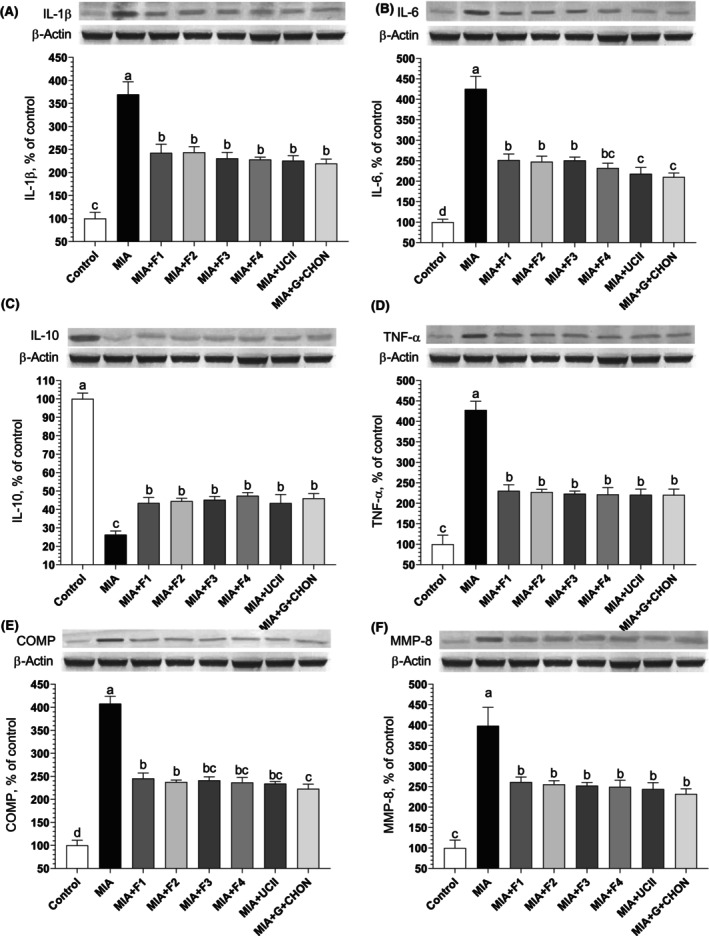
Effects of different joint health formulations on knee joint protein expression of IL‐1β (A), IL‐6 (B), IL‐10 (C), TNF‐α (D), COMP (E), and MMP‐8 (F), levels in monosodium iodoacetate (MIA)‐induced OA rats. The densitometric analysis of the relative intensity according to the control group of the Western blot bands was performed with β‐Actin normalization to ensure equal protein loading. Blots were repeated at least three times (*n* = 3), and a representative blot is shown. The bars and error lines point out the mean and standard deviation. ANOVA and Tukey's post hoc test were performed for statistical comparison. Different letters (a, b, and c) above the bars indicate statistical differences among the groups (*p* < .05). IL‐1β, interleukin‐1β; IL‐6, interleukin‐6; IL‐10, interleukin‐10; TNF‐ α, tumor necrosis factor α; COMP, cartilage oligomeric matrix protein; MMP‐8, matrix metalloproteinase‐8. F1: Curcuminoids+Gingerols+acetyl‐11‐keto‐β boswellic acid (AKBA); F2: Curcuminoids+Withania Glycosides+ AKBA; F3: Curcuminoids+Total Withanolides+AKBA; F4: Curcuminoids+AKBA; UCII: Undenatured type II collagen; G + CHON: Glucosamine Hydrochloride, Chondroitin Sulfate, Hyaluronic Acid, Calcium Fructoborate.

The most important class of compounds contributing to the pathophysiology of OA is the group of inflammatory cytokines, with IL‐1𝛽, TNF, and IL‐6 playing the most significant roles (Wojdasiewicz et al., 2014). Rat synovial tissue protein levels were assessed using the Western blot technique. Rats with MIA‐induced OA had considerably higher levels of IL‐1𝛽, TNF, and IL‐6 than the control group; however, treatment with the OAHT formulations caused this level to drop favorably (Figure 7A–C). In accordance with other parameters, curcuminoids + AKBA (MIA + F4 group) decreased IL‐1𝛽 levels by 38.26%, TNFα levels by 48.22%, and IL‐6 levels by 45.41% in synovial tissue compared to untreated OA rats. However, when MIA + F1–F4 were compared, no significant difference was observed in synovial tissue IL‐1𝛽, TNFα, and IL‐6 levels (*p* > .05).

Plant‐based anti‐inflammatory compounds can induce the expression and production of IL‐10 and increase its effect on multiple tissues (Mollazadeh et al., 2019). Anti‐inflammatory cytokine IL‐10 levels were significantly lower in MIA‐induced OA rats compared to the control group. Specifically, curcuminoids + AKBA (MIA + F4) increased the level of the anti‐inflammatory marker IL‐10 in the synovial tissue of rats by 44.62% compared with the OA untreated group (*p* < .0001; Figure 7D). However, there was no significant difference between the groups treated with the OAHT formulations and those that received UCII and G + CHON (*p* > .05).

Similarly, compared to controls, synovial tissue MMP‐8 and COMP levels were higher in rats with MIA‐induced OA. Administration of the formulations for 4 weeks after MIA injection decreased synovial tissue MMP‐8 and COMP levels, indicating a beneficial effect on cartilage catabolism as in similar studies (Kullich et al., 2002). Comparing the supplements, especially curcuminoids + AKBA (MIA + F4), with the OA untreated group, there was a 41.99% reduction in COMP levels and a 37.51% reduction in MMP‐8 levels (Figure 7E,F). However, no significant difference was observed when the F1‐F4 formulations and UCII and G + CHOND were compared, and values close to the positive controls emerged (*p* > .05). The OAHT supplements effectively increased IL‐10 levels while decreasing protein levels of IL‐1β, IL‐6, TNF‐α, COMP, and MMP‐8 in the synovial tissue of rats with MIA‐induced OA. Thus, it was determined that all the formulations that were applied alleviated the severity of OA.

## 4 DISCUSSION

Osteoarthritis (OA), the most common degenerative joint condition, particularly prevalent among older individuals, stands as the most prominent synovial joint ailment that leads to intense pain and structural damage. From a pathological perspective, OA is marked by synovial inflammation, the formation of marginal osteophytes, sclerosis in the subchondral bone, and the deterioration of articular cartilage. The chronic pain associated with OA poses a significant challenge, as existing treatments are inadequate in addressing it (Dara et al., 2023; Li et al., 2021).

This study evaluated the anti‐OA effects of plant‐based F1–F4 in MIA‐induced OA rats. These formulations, containing bioactive ingredients from herbs, were observed to alleviate the severity of OA and positively influence the development of OA by reducing oxidative stress, inflammation, and joint damage. The constituents of these treatments are known for their various health benefits. Additionally, similar anti‐OA findings were obtained in previous rat studies conducted at our institute using the MIA‐induced knee OA model, which had been standardized in prior research (Orhan et al., 2022; Sahin et al., 2021; Yabas et al., 2021).

As a metabolic inhibitor, MIA inhibits the glyceraldehyde‐3 phosphate dehydrogenase enzyme, disrupting cell glycolysis (Abo‐Zalam et al., 2021). Subsequently, this increases oxidative stress in the environment, resulting in chondrocyte loss and reduced cartilage thickness and osteolysis. These changes produce histological and morphological alterations in the articular cartilage, similar to those seen in OA patients (Kara et al., 2021; Liu et al., 2021). The current study evaluated joint swelling as an inflammation marker after OA induction with MIA. The right knee joint diameter was significantly augmented in OA rats, with an increased inflammatory response. However, these values decreased significantly after the administration of F1–F4 formulations. Additionally, the discomfort associated with knee OA worsens over time, and persons with the condition tend to walk at a characteristic pace to cope with the pain. It has been suggested that the stride length and walking speed of OA patients are reduced (Robbins et al., 2016; Yabas et al., 2021). The plant‐based formulations applied in our study significantly increased the stride length and paw area linearly with their antioxidant potential and anti‐inflammatory effect, positively impacting gait abnormalities caused by OA. Based on study data, OA rats treated with plant‐based F1‐F4 formulations showed improved histopathology of the knee joint and decreased joint abnormalities according to the Kellgren–Lawrence classification (Kellgren & Lawrance, 1957; Table 2). Choudhary et al., in a study of rats with MIA‐induced OA, concluded that the application of *Spinacia oleracea* extract could alleviate the effects of arthritis (Choudhary et al., 2018). In a mouse model of MIA‐induced OA in the knee, a preparation used in traditional Indian medicine (Dashmoolarishta) consisting of ten different herbal mixtures improved the joint architecture deterioration known to occur with OA, similar to our study (Shetty et al., 2017).

In the present study, cartilage was damaged when the balance was disturbed due to impaired metabolism of chondrocytes, synoviocytes, and subchondral bone cells, resulting in increased Mankin scores similar to previous reports (Lu et al., 2021). The OAHT preparations possibly strengthened the cartilage integrity and eliminated the structural defects, significantly reducing Mankin scores. The turmeric treatment applied by Jin et al. in OA rats with MIA showed lower Mankin scores in both the femur and tibia compared to an untreated OA group (Jin et al., 2022). It is thought that the anti‐OA function of curcumin, an essential component of F1‐F4 formulations in our current study, is also associated with increased collagen anabolism and decreased inflammatory catabolism in articular cartilage.

Recent studies have shown that the concentrations of COMP and CRP are reliable factors for monitoring cartilage damage and determining the therapeutic response. These biomarkers are strongly associated with articular cartilage degradation (Saghafi et al., 2017; Zhang, 2018). Previous studies in OA have pointed to the use of serum and muscle COMP as a marker for early cartilage lesions in the knee, which correlate negatively with disease duration (Chandran et al., 2019). A study on OA rats reported that serum COMP and CRP levels of OA rats receiving a new‐generation curcumin therapy were significantly suppressed compared to an OA untreated group (Yabas et al., 2021). In the current study, CRP (serum) and COMP (serum and muscle tissue) levels were increased in OA rats, but the situation was reversed, with CRP and COMP levels significantly decreasing in rats treated with F1‐F4 formulations. Consistent with these findings, it was found that F1 to F4 was responsible for significantly improving OA pathogenesis in rats via modulation of proinflammatory cytokines. Thus, it was determined that F1‐F4 positively affects numerous pathways, including OA pathogenesis mediators, such as COMP and CRP, and alleviates overall disease.

Apoptosis of chondrocytes is one of the leading causes of OA and can be explained by oxidative stress. Previous studies have shown that oxidative stress is associated with chronic inflammatory diseases and plays a vital role in the physiology and pathophysiology of OA (Chen et al., 2019). At the same time, OA causes the accumulation of ROS and increases MDA production in chondrocytes (Guo et al., 2022). Like other mammalian cells, chondrocytes contain several antioxidant defenses against the adverse effects of ROS. Antioxidants can neutralize ROS, stop their creation, or reverse the harm they have already caused. Among these protective mechanisms is a coordinated system of antioxidant enzymes produced by CAT, SOD, and GSH‐Px (Henrotin & Kurz, 2007). In the present study, serum antioxidant enzyme levels decreased in OA rats, and the amount of MDA, an essential marker of lipid peroxidation, increased. Consistent with the present study, Jangravi et al. found that the avocado/soybean unsaponifiables compound reduced MDA in OA patients. In addition, when antioxidant status was evaluated by measuring total antioxidant capacity, SOD, CAT, and GSH‐Px levels, they found that, unlike MDA, the amount of all antioxidants increased after consumption of avocado/soybean unsaponifiable matter (Jangravi et al., 2021). Therefore, it is assumed that F1‐F4 supplements increase the levels of antioxidant enzymes due to the potent antioxidant properties of the herbal compounds in the formulation.

It is known that proinflammatory cytokines play essential roles in the pathogenesis of OA by inducing synovial inflammation that leads to cartilage destruction (Jo et al., 2020). Abnormally high levels of IL‐1β, IL‐6, and TNF‐α in OA patients have been recognized as an important factor contributing to cartilage loss in these individuals (Wang & He, 2018). In addition, IL‐10 deregulation plays a role in developing many inflammatory diseases, such as neuropathic pain and OA (Mollazadeh et al., 2019). In the study, IL‐1β, IL‐6, and TNF‐α levels in serum and knee joint tissues increased in the OA group compared to the control group, while IL‐10 levels, which have anti‐inflammatory properties, were decreased in the knee joint tissue. Similar to the data obtained in the current study, in a clinical study conducted by Yang et al. on OA patients, it was determined that plant‐derived vitexin inhibited IL‐1β, IL‐6, and TNF‐α expressions in serum and synovial tissue in the treated patients (Yang et al., 2019). Proinflammatory cytokines in serum and knee joint tissue were significantly decreased in rats that received OAHT formulations compared to the OA untreated group. In contrast, the level of the anti‐inflammatory cytokine IL‐10 increased significantly in knee joint tissue. Thus, it is estimated that in rats with OA, the herbal ingredients modulate cytokines, reduce inflammation, and exert a chondroprotective effect.

Production of MMPs that degrade the cartilage matrix under inflammatory conditions has been suggested to increase the degradation of the extracellular matrix (Luo et al., 2021). MMP‐8, a member of the MMP family, is believed to be closely associated with OA‐induced cartilage destruction, as confirmed by human and animal studies (Wu et al., 2014). In our study, the knee joint tissue MMP‐8 level increased in the OA group compared to the control group, and it decreased significantly in the groups in which F1‐F4 formulations were applied, showing a reducing effect on cartilage destruction. MMP‐2, MMP‐8, and MMP‐9 synthesis increased in rat chondrocytes stimulated by MIA in an in vivo study similar to the current work. Treatment with luteolin, a plant flavonoid, has been observed to inhibit the upregulation of these IL‐1‐dependent MMPs (Fei et al., 2019). These findings imply that inhibition of these MMPs may be responsible for the anticatabolic action of F1‐F4 given to rats with OA.

An important question arising from our results is how F1‐F4 supplements reduce OA severity and pathophysiology in rats. Curcumin has been shown to decrease OA inflammation in cell cultures, animal models, and human investigations. In cultured human chondrocytes, curcumin reduced IL‐1‐induced cell death, apoptosis, and the production of IL‐6, IL‐8, TNF‐α, PGE2, ICAM‐1, and COX‐2 (Nicoliche et al., 2020). In the future, we may focus further on curcumin's function in OA. More phenolic pigments are present in turmeric (including curcumin, dethoxycurcumin, and bisdemethoxycurcumin). These components may show a better clinical effect by increasing their pharmacokinetic concentrations in the blood of OA patients and their residence time in the body. According to recent research, the bioavailability of curcumin molecule monomers is limited (Zeng et al., 2021). In addition, gingerol may block the formation of lipid peroxidation, depletion of antioxidant status, and NF‐kB‐mediated inflammatory responses (Abusarah et al., 2017). Additionally, ginger extract is recognized as an effective anti‐inflammatory agent in the treatment of rheumatoid arthritis and OA (Mohd Sahardi & Makpol, 2019). In a clinical OA investigation, 1 g of ginger per day was found to lower TNF‐α and IL‐1β, two inflammatory cytokines that can activate the lipoxygenase pathway (Mozaffari‐Khosravi et al., 2016). In addition, it has been reported that various ailments such as OA, multiple sclerosis, and Crohn's disease have been successfully treated with Boswellia extracts and phytochemicals (Efferth & Oesch, 2022). The percentage of the AKBA component is crucial to improving the Boswellia product's anti‐inflammatory potential. Notably, in Freund's rat model of adjuvant‐induced arthritis, demonstrated noticeably more decisive anti‐inflammatory action than regular Boswellia extract containing 3% AKBA (Sengupta et al., 2011). *Withania somnifera* (Ashwagandha) extract, the plant from which the components of the formulations used in the study were obtained, is an anti‐inflammatory and anti‐arthritic agent found to be beneficial in clinical cases of rheumatoid and OA (Singh et al., 2011). The key finding was tested in an explant model of human OA cartilage damage, where *W. somnifera* root powders showed short‐term chondroprotective activity in 50% of OA cases (Sumantran et al., 2007). It is postulated that our study results are due to the formulations' anti‐inflammatory, antioxidant, and anti‐arthritis properties of the active ingredients (curcuminoids, gingerols, AKBA, Withania glycosides, total withanolides).

## 5 CONCLUSION

In conclusion, these results suggest that a novel, multi‐ingredient formulation, F1‐F4, can reduce the severity of MIA‐induced OA in rats. This effect may be mediated by altering the levels of inflammatory mediators and oxidative stress indicators. Moreover, it has been confirmed that the herbal‐based formulations applied in the study show similar effects as UCII and G + CHON, whose benefits are known. In addition to conventional therapies, OAHT formulations might provide a reasonable alternative for treating clinical OA, which should be confirmed by meticulously planned research. As a limitation of our work, human studies may compare OAHT with NSAIDs and corticosteroids frequently used to treat OA symptoms. Although the MIA model of OA is commonly used to research the pathophysiology of OA, it should be highlighted that it has some drawbacks, including a faster rate of OA development in the animal model than in humans. Clinical trials conducted over a long period are required to clarify how supplements enhance functional status in OA patients.

## AUTHOR CONTRIBUTIONS

**Fusun Erten:** Data curation (equal); formal analysis (equal); methodology (equal). **Oguzhan Ozdemir:** Data curation (equal); formal analysis (equal); methodology (equal); writing – original draft (equal). **Muhammed Tokmak:** Data curation (equal); formal analysis (equal); methodology (equal). **Ali Said Durmus:** Formal analysis (equal); methodology (equal). **Ibrahim Hanifi Ozercan:** Formal analysis (equal); methodology (equal). **Abhijeet Morde:** Writing – original draft (equal); writing – review and editing (equal). **Muralidhara Padigaru:** Writing – original draft (equal); writing – review and editing (equal). **Kazim Sahin:** Conceptualization (equal); funding acquisition (lead); investigation (lead); methodology (lead); project administration (lead); writing – original draft (equal); writing – review and editing (equal).

## FUNDING INFORMATION

This project was funded by OmniActive Health Technologies (Mumbai, India) and the Turkish Academy of Sciences (TUBA, KS) in part. The funders were not involved in the project design, collection, analysis, and interpretation of data, the writing of this article, or the decision to submit it for publication.

## CONFLICT OF INTEREST STATEMENT

Abhijeet Morde and Muralidhara Padigaru. are employees of OmniActive Health Technologies (Mumbai, India). The remaining authors declare that the research was conducted in the absence of any commercial or financial relationships that could be construed as a potential conflict of interest.

## Data Availability

Correspondence and requests for materials should be addressed to K. Sahin.
